# Comparison of different electrocardiographic criteria for the detection of previous myocardial infarction in relation to infarct characteristics as assessed with cardiovascular magnetic resonance imaging

**DOI:** 10.1186/1532-429X-15-S1-E70

**Published:** 2013-01-30

**Authors:** Caroline Jaarsma, Sebastiaan C Bekkers, Zaki Haidari, Martijn W Smulders, Patricia J Nelemans, Anton Gorgels, Harry J Crijns, Joachim E Wildberger, Simon Schalla

**Affiliations:** 1 Cardiology, Maastricht University Medical Center, Maastricht, Netherlands; 2 Radiology, Maastricht University Medical Center, Maastricht, Netherlands; 3 Cardiovascular Research Institute Maastricht (CARIM), Maastricht University, Maastricht, Netherlands; 4 Epidemiology, Maastricht University, Maastricht, Netherlands

## Background

The electrocardiogram (ECG) is often used as initial test to detect or rule out previous myocardial infarction (MI). Although different ECG-criteria with considerable heterogeneity are used for this purpose, their accuracy is not well described. We aimed to determine the diagnostic accuracy of four commonly used ECG-criteria for detecting previous MI: 1) the universal definition of previous MI, 2) the Minnesota ECG-code (MC), 3) the Selvester QRS-score, and 4) assessment by cardiologists, using delayed-enhancement cardiovascular magnetic resonance imaging (DE-CMR) as reference standard. Also the effect of different ECG and infarct characteristics on detecting previous MI were evaluated.

## Methods

The 3-month follow-up ECGs of 78 first, reperfused ST-elevation MI (STEMI) patients were pooled with ECGs of 36 healthy controls. All 114 ECGs were randomly analyzed, blinded to clinical and DE-CMR data. Sensitivity, specificity, and diagnostic accuracy were determined for the universal definition, MC, Selvester QRS-score, and visual assessment by two cardiologist with >10 years of clinical experience. DE-CMR (104±11 days post MI) showed hyperenhancement matching the infarct-related artery (IRA) territory in all patients. The effect of ECG patterns and infarct characteristics on probability of MI detection was evaluated using logistic regression analysis. For anterior MI (LAD 31%), leads I, aVL, V1-V6 and for non-anterior MI (LCx 14%, RCA 55%) leads II, III, aVF, V1-V2 were evaluated.

## Results

**Figure 1:**
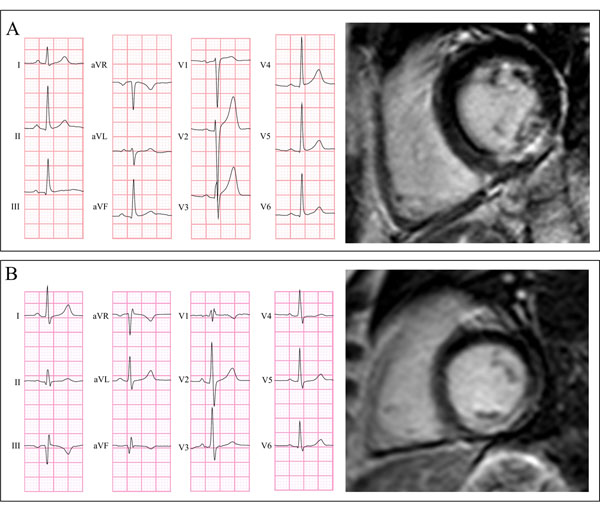
Electrocardiogram and corresponding delayed-enhancement cardiovascular magnetic resonance short axis view of an undetected (A) and a detected (B) myocardial infarction. Electrocardiogram (ECG) and corresponding cardiovascular magnetic resonance delayed-enhancement short axis view of a 58-year-old man with an LCx-related myocardial infarction (infarct size 8.5% of the left ventricle and 65% transmural) that was not detected by any of the ECG-criteria (A) and a 55 year-old man with an LCx-related myocardial infarction (infarct size 7.5% of the left ventricle and 51% transmural) that was detected by all four ECG-criteria (B).

The sensitivity of the universal definition, MC, Selvester QRS-score, and cardiologists was 33%, 79%, 88%, and 67%, specificity was 97%, 72%, 31%, and 89%, diagnostic accuracy was 54%, 77%, 70%, and 74%, respectively (Figure 1). Probability of detection of MI by cardiologists increased with an increasing number (odds ratio [OR] 2.00 (95% confidence interval [CI] 1.30-3.09)), width (OR 1.02 (95%CI 1.01-1.03)), and depth (OR 1,16 (95%CI 1.07-1.27)) of Q-waves in anterior MI and Q- or R-waves in non-anterior MI (p<0.01 for all). Increasing infarct size and transmurality also increased the likelihood of detecting previous MI (OR 1.15 (95%CI 1.06-1.25), p<0.01, and OR 1,05 (95%CI 1.01-1.08), p=0.01). Five infarctions (6%) were not detected by any of the ECG-criteria (infarct size and transmurality ranging from 1.5-8.5% and 32-86%, respectively).

## Conclusions

For detecting previous MI, the time-consuming MC and visual assessment by cardiologists achieved the best and similar diagnostic accuracies. The likelihood of detecting previous MI increased with an increasing number, depth, and width of Q-waves in anterior MI and Q- or R-waves in non-anterior MI, as well as increasing infarct size and transmurality. However, a considerable number of infarctions remain undetected by all ECG-criteria.

## Funding

None.

